# Implant Plane Does Not Seem to Impact Shoulder Function after Direct-to-Implant Breast Reconstruction in Non-Radiated Patients: A Randomized Controlled Trial

**DOI:** 10.1055/a-2662-2283

**Published:** 2025-09-18

**Authors:** Signe von Buchwald, Diana Lydia Dyrberg, Farima Dalaei, Jens Ahm Sørensen, Jørn Bo Thomsen

**Affiliations:** 1 Department of Plastic Surgery, Research Unit for Plastic Surgery, Odense University Hospital, Odense C, Denmark

**Keywords:** impaired shoulder function, functional assessment, subpectoral implant placement, prepectoral implant placement, direct-to-implant reconstruction

## Abstract

**Background:**

Subpectoral implant placement in breast reconstruction has been associated with potential shoulder function impairment compared to prepectoral placement, though the evidence remains inconclusive. This study aimed to investigate differences in shoulder function following mastectomy and direct-to-implant breast reconstruction using prepectoral or subpectoral implant placement.

**Methods:**

Forty-two women aged 18 years or older, eligible for direct-to-implant breast reconstruction, were randomized 1:1 to undergo either prepectoral or subpectoral implant placement. Data were collected at baseline and at 3- and 12-month follow-ups. Shoulder function was assessed using the validated Constant Shoulder Score (CSS), which evaluates pain, activities of daily living, range of motion, and strength. Pectoralis major muscle (PMM) strength was also measured.

**Results:**

Baseline and demographic characteristics were comparable between the groups. No differences were observed in total CSS or the modified CSS (including the PMM strength) between the prepectoral and subpectoral groups at baseline or at the 3- and 12-month follow-ups. At 12 months, total CSS ( *p* = 0.74) and modified CSS ( *p* = 0.45) remained similar across both groups.

**Conclusion:**

There were no significant differences in shoulder and arm function between the sub- and prepectoral reconstruction groups. These findings suggest that concerns regarding shoulder function should not dictate the choice of implant placement plane in direct-to-implant breast reconstruction.

## Introduction

Direct-to-implant breast reconstruction can be performed following nipple or skin-sparing mastectomy using different implant pocket planes, provided that no radiation to the breast area has been administered or is planned. 1 2 Traditionally, subpectoral implant placement has been the preferred technique due to concerns that prepectoral implant placement increases the risk of complications such as hematoma, capsular contracture, and rippling. 3 4 However, the advent of dermal matrices has facilitated the adoption of prepectoral implant placement, making it a viable option for direct-to-implant breast reconstruction. While subpectoral placement has long been favored, it is associated with breast animation deformity, higher postoperative pain and potentially impaired shoulder function, prompting growing interest in the prepectoral technique. 2 5 6 7 However, the functional outcomes associated with each pocket plane remain insufficiently understood, highlighting the need for further investigation into their respective advantages and disadvantages. 8

Prospective and retrospective studies have evaluated shoulder function in patients reconstructed by subpectoral implant placement. 6 7 9 10 However, high-quality reproducible data are needed to evaluate and compare shoulder function and pectoralis major muscle (PMM) strength in patients following pre- and subpectoral implant placement after breast reconstruction. Systematic reviews assessing the postoperative complications in pre- and subpectoral breast reconstruction highlight a lack of randomized controlled trials (RCTs) in the literature, 4 emphasizing the need for well-designed (RCTs) with adequate follow-up periods 11 and strong baseline comparisons 12 to evaluate outcomes such as pain and shoulder function. This study reports the secondary outcomes of an RCT comparing shoulder function and PMM strength in women undergoing direct-to-implant breast reconstruction with either prepectoral or subpectoral implant placement. The primary outcome of the trial focused on breast animation deformity.

## Methods

The data presented in this paper were collected as secondary outcome measures as part of an RCT. The primary outcome measure of the trial was the occurrence of breast animation deformity in patients following mastectomy and direct-to-implant breast reconstruction. The protocol and trial have been published and include a detailed description of the materials and methods. 13 14 The study was approved by the Danish Committee on Health Research Ethics for Southern Denmark outside Europe; this approval is often referred to as the institutional review board number (S-20160160) and the Danish Data Protection Agency (17/13640) and reported according to the principles of the Declaration of Helsinki. The original study was registered on clinicaltrials.gov (NCT03143335).

This multicenter RCT was designed as a superiority trial with two parallel study arms and was conducted in accordance with the CONSORT guidelines. 15 The study included 42 patients undergoing therapeutic or prophylactic nipple or skin-sparing mastectomy, followed by direct-to-implant breast reconstruction. Patients were randomized in a 1:1 ratio to receive either prepectoral or subpectoral implant placement. Two Departments of Plastic Surgery participated in the trial. Enrollment commenced from April 2017 and ended in March 2020.

The inclusion criterion for the study was women aged 18 years or older eligible for direct-to-implant breast reconstruction following mastectomy. Exclusion criteria included tobacco use, hypertension requiring treatment with more than one medication, a body mass index (BMI) <22 or >32 kg/m ^2^ , pre- or postsurgical radiotherapy to the breast, breast ptosis >2 as assessed by Regnault's ptosis scale, 16 a psychiatric diagnosis, or dementia that could impair the ability to provide informed consent. 13 The patients were screened for eligibility by a consultant plastic surgeon in the outpatient clinic. Patients meeting the criteria were invited to participate and given time for reflection before giving informed consent. After informed and written consent was obtained, patients were enrolled in the study. Final eligibility was confirmed during surgery, based on the condition of the mastectomy flaps. Participants were randomized to either of the two intervention groups, with bilateral cases treated as a single case. Neither patients nor investigators were blinded after surgery. The patients were examined in the outpatient clinic the day before reconstruction, with follow-ups at 3 and 12 months postoperatively. Demographic and clinical data were recorded, including age, BMI, indication for mastectomy, laterality of reconstruction, duration of surgery, implant specifications, chemotherapy status, and length of follow-up.

### Surgical Procedures

**Fig. 1:**
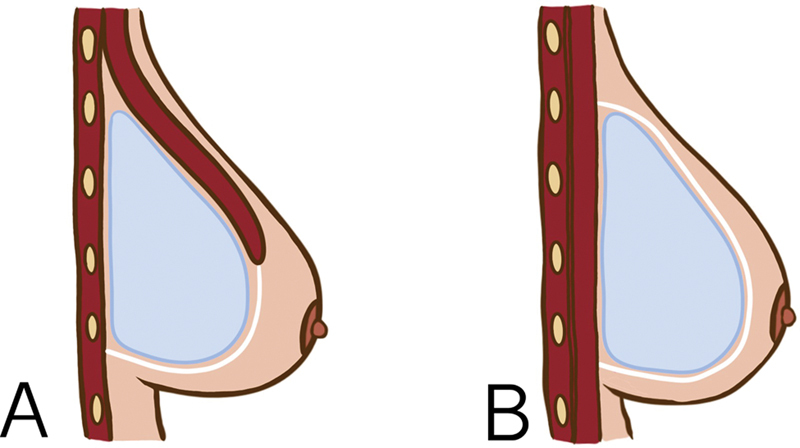
Direct-to-implant reconstruction surgical procedures. **(A)** Subpectoral implant placement. **(B)** Prepectoral implant placement.

Direct-to-implant breast reconstructions were performed following nipple or skin-sparing mastectomies. The procedures were performed by a total of eight surgeons. Mastectomies were conducted by four breast surgeons and reconstructions by four plastic surgeons. To ensure uniformity, procedures were carried out with a standardized technical approach. The surgical method for the nipple and skin-sparing mastectomy has been described in previous studies. 17 18 The mastectomy flaps were evaluated by the surgeon after the mastectomy to determine their quality prior to reconstruction. Patients with thin flaps deemed to have poor vascularity or at risk for poor outcomes were excluded from the study. Eligible patients were randomized to undergo reconstruction with either prepectoral or subpectoral implant placement. When placing the implant in a subpectoral pocket, the PMM was released from its inferomedial insertion to provide superior support and coverage of the implant ( Fig. 1A ). 1 To ensure inferior support, one piece of acellular dermal matrix (Meso BioMatrix®, 10 × 16 cm; AMM) was used and sutured to the inferior crease of the PMM and the serratus fascia, creating a hammock for the implant. The PMM was left undisturbed in prepectoral reconstruction, and the implant was placed above the muscle. Support was provided by two pieces of AMM, which were sutured circumferentially to the PMM ( Fig. 1B ). 1 Regardless of the surgical technique used, all patients followed a similar postoperative care protocol. All the patients were guided by a physiotherapist to optimize the postoperative shoulder and arm rehabilitation. As standard the physiotherapy was initiated before discharge while the patients were still hospitalized.

### Constant Shoulder Score

The shoulder function was assessed by the validated Constant Shoulder Score (CSS), recommended by the European Society for Surgery of the Shoulder and Elbow and the Journal of Shoulder and Elbow Surgery. 19 Assessments were made in the outpatient clinic the day before surgery, and 3 and 12 months after surgery. Each patient had the function of their shoulder and upper arm as well as their PMM strength assessed bilaterally. We used a previously developed standardized CSS test protocol. 20 This protocol is based on the newest and modified CSS guidelines provided by Constant et al. in 2008. 19 The CSS evaluates two subjective parameters, pain, and activities of daily living (ADLs), and two objective parameters, the range of motion (ROM) and strength. The test enables a maximum score of 100 points, for a young, healthy individual, distributed by 35 points for subjective assessment and 65 points for objective measures ( Table 1 ). Pain assessment was based on the highest level of pain experienced by the patient during ordinary activities over a period of 24 hours and visualized on an analog scale on paper. The ADL assessment consisted of four questions dealing with everyday activities experienced over the preceding week, including sleep disturbance, daily work, recreational activities, and to which level the hand could be used comfortably. ROM, in internal and external rotations, was tested by asking the patients to perform different movements painlessly and without help. For testing ROM in flexion and abduction, we used a goniometer, while the strength was assessed using a dynamometer (IsoForceControl® EVO2, MDS Medical Device Solutions AG, Switzerland). Each patient was assessed in a standardized manner and in accordance with the guidelines of the test. When assessing the strength of the PMM, each patient was placed on a bench in the supine position. Using a dynamometer with the suction disc applied to the floor, the patient was asked to push the dynamometer's handle toward the ceiling with the elbow bent at 90 degrees. The patient was instructed to push upwards for 5 seconds with maximal effort while being verbally encouraged. The score was calculated from the highest score of three attempts. The PMM strength measures were added to the total CSS, referred to as the modified CSS.

**Table 1:** Visualization of the parameters included in the total Constant Shoulder Score and modified Constant Shoulder Score

Visualized CSS		Parameters	Assessment	Types of measures	Point distribution
Modified CSS	Total CSS	Subjective parameters	Pain	Visual analog scale	0–15
			ADL	Four questions regarding everyday activities	0–20
		Objective parameters	ROM	Four different shoulder movements	0–40
			Strength	Measured by dynamometer	0–25
				Total CSS score	0–100
		Additional parameter	PMM strength	Measured by dynamometer	Force generated
				Total modified CSS score	Total CSS + PMM strength

Abbreviations: ADL, activity of daily living; CSS, Constant Shoulder Score; PMM, pectoralis major muscle; ROM, range of motion.

### Statistical Analysis

Baseline variables were used to describe the characteristics of the participants. All data were typed in by double data entry in the REDCap Database System. 21 We used mean and standard deviation for continuous variables, and numbers and percentages for categorical variables. Repeated measures with no adjustment and an independent samples *t* -test were used to compare the results of shoulder function and PMM strength assessments within and between groups, respectively. Repeated measures analysis was deemed necessary in this study to evaluate changes within the same individuals pre- and postoperatively, ensuring that intraindividual variations were accounted for, and statistical power was improved by reducing between-subject variability. A two-sided *p* -value <0.05 was considered significant. For statistical analysis, we used IBM SPSS Statistics version 28.0 (IBM Corporation, Armonk, NY).

## Results

**Fig. 2:**
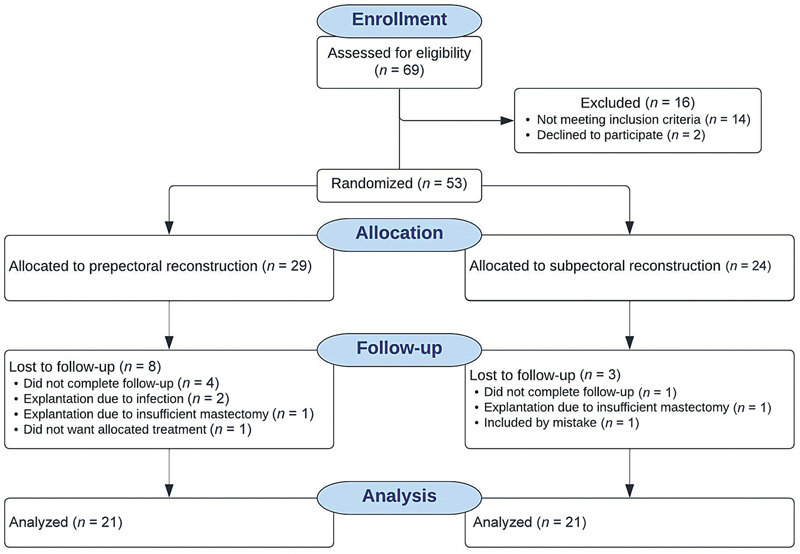
Flowchart showing enrollment, allocation, follow-up, and analysis of included patients.

Between April 2017 and March 2020, a total of 69 women were screened for eligibility, of which 53 met the inclusion criteria ( Fig. 2 ). Sixteen of the 69 women were excluded from the study as they did not meet the inclusion criteria ( *n* = 14) or did not wish to participate ( *n* = 2). The exclusion causes have previously been described in detail. 13 A total of 53 patients were included in the study, with 29 allocated to the prepectoral group and 24 to the subpectoral group. A total of 11 patients were lost to follow-up, distributed as 8 patients in the prepectoral group and 3 in the subpectoral group. This resulted in a total of 21 allocated to each intervention group for analysis ( Fig. 2 ).

Due to missing data at 12-month follow-up, one patient was not included in the strength and total CSS results on either side, and one patient was not included in the ROM, strength, and total CSS results on their left side. The right-side data were retained as this patient was bilaterally reconstructed.

Patients' descriptive statistics have been reported and described previously. 13 In brief, the mean age was 49.4 ± 10.9 and 50.0 ± 10.2 ( *p* = 0.84) for the prepectoral and subpectoral group, and the BMI was 25.5 ± 2.4 and 26.8 ± 2.25 ( *p* = 0.083), respectively. A total of 60 reconstructed breasts were included in the trial. In the prepectoral and subpectoral groups, the mastectomies were therapeutic in 61% and 67% of the cases, respectively. Furthermore, 43% and 71% of patients had unilateral surgery, whereas 57% and 29% had bilateral surgery, respectively. A total of 27 breasts (45%) were reconstructed with subpectoral implant placement and 33 breasts (55%) with prepectoral placement. No patients received radiotherapy to the breast or underwent axillary dissection, but a total of 16 patients did receive chemotherapy as part of their breast cancer treatment. The two groups were comparable and showed no differences regarding the descriptive data. 13

**Fig. 3:**
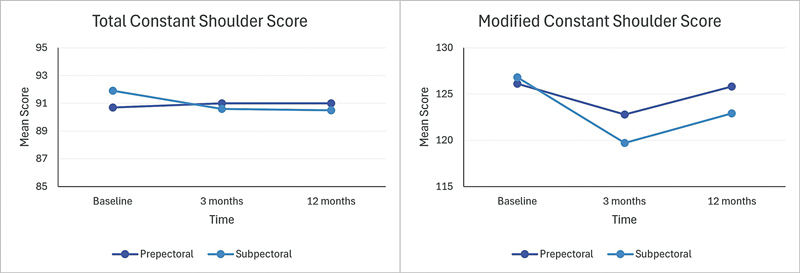
Depiction of the total CSS and the modified CSS in the prepectoral and subpectoral groups. CSS, Constant Shoulder Score.

Comparing the prepectoral and the subpectoral group in regard to total CSS and modified CSS, we found no significant differences at baseline or follow-up at 3 and 12 months ( Table 2 and Fig. 3 ). An intragroup analysis comparing the total CSS and modified CSS in each group at baseline with 3- and 12-month follow-up showed no significant changes in in total CSS for the prepectoral group. Notably, significant differences emerged in the subpectoral group and the modified CSS in the prepectoral group at 3-month follow-up, although this difference did not persist, as neither reconstructed group showed a significant difference in shoulder function at 12-month follow-up ( Table 3 ). Comparing the submeasures of the preprectoral and subpectoral group showed no significant difference ( Table 4 ). However, an intragroup analysis examining the submeasures showed a significant difference in PMM strength at 3-month follow-up in both groups. Though these differences did not persist at the 12-month follow-up either. Additionally, the analysis showed that pain differed significantly in the subpectoral group at 12-month follow-up compared to baseline. The ROM, ADLs, and strength showed no significant differences ( Table 5 ).

**Table 2:** Constant Shoulder Score tested in the prepectoral and subpectoral group preoperatively (baseline), and at 3 and 12 months postoperatively

Constant Shoulder Score		Prepectoral, mean ± SD	Subpectoral, mean ± SD	*p* -Value
Total CSS	Baseline	90.7 **±** 3.3	91.9 **±** 4.0	0.182
	3 months	91.0 **±** 4.4	90.6 **±** 4.4	0.735
	12 months	91.0 **±** 5.2	90.5 **±** 6.3	0.744
Modified CSS	Baseline	126.1 **±** 10.8	126.8 **±** 10.4	0.806
	3 months	122.8 **±** 10.6	119.7 **±** 11.3	0.281
	12 months	125.8 **±** 13.5	122.9 **±** 14.6	0.453

Abbreviations: CSS, Constant Shoulder Score; SD, standard deviation.

**Table 3:** The baseline data of total Constant Shoulder Score and modified Constant Shoulder Score compared with data from 3- and 12-month follow-up of the same group

Intragroup analysis		Baseline: 3 months, *p* -value	Baseline: 12 months, *p* -value
Prepectoral	Total CSS	0.599	0.696
	Modified CSS	**0.043**	0.885
Subpectoral	Total CSS	**0.043**	0.169
	Modified CSS	**<0.001**	0.073

Abbreviation: CSS, Constant Shoulder Score.

**Table 4:** Submeasures tested in the prepectoral and subpectoral group preoperatively (baseline), and at 3 and 12 months postoperatively

Submeasures		Prepectoral mean ± SD	Subpectoral mean ± SD	*p* -Value
Pain (0–15)	Baseline	14.9 ± 0.5	14.9 ± 0.4	0.890
	3 months	14.9 ± 0.3	14.7 ± 1.1	0.250
	12 months	14.8 ± 0.7	14.0 ± 2.5	0.062
ADL (0–20)	Baseline	19.9 ± 0.3	20.0 ± 0.0	0.112
	3 months	19.9 ± 0.5	19.9 ± 0.3	0.933
	12 months	19.6 ± 1.0	19.5 ± 2.3	0.792
ROM (0–40)	Baseline	40.0 ± 0.0	40.0 ± 0.0	1.000
	3 months	40.0 ± 0.0	40.0 ± 0.0	1.000
	12 months	40.0 ± 0.0	40.0 ± 0.0	1.000
Strength (0–25)	Baseline	15.8 ± 3.3	17.0 ± 4.0	0.222
	3 months	16.1 ± 4.2	16.0 ± 3.8	0.877
	12 months	16.6 ± 4.5	17.1 ± 4.5	0.672
PMM strength (∞)	Baseline	35.4 ± 8.3	34.8 ± 7.3	0.772
	3 months	31.8 ± 7.2	29.1 ± 7.9	0.171
	12 months	34.5 ± 8.8	32.4 ± 9.4	0.388

Abbreviations: ADL, activity of daily living; PMM, pectoralis major muscle; ROM, range of motion; SD, standard deviation.

**Table 5:** The baseline data of all submeasures compared with data from 3- and 12-month follow-up of the same group

Submeasures, *p* -values		Pain	ADL	ROM	Strength	PMM strength
Prepectoral	Baseline: 3 months	0.808	0.721	1.000	0.676	**0.008**
	Baseline: 12 months	0.754	0.357	1.000	0.348	0.546
Subpectoral	Baseline: 3 months	0.112	0.239	1.000	0.330	**<0.001**
	Baseline: 12 months	**0.004**	0.144	1.000	0.749	0.188

Abbreviations: ADL, activity of daily living; PMM, pectoralis major muscle; ROM, range of motion.

## Discussion

This RCT assessed shoulder and upper arm function as a secondary outcome measure in 42 women undergoing nipple or skin-sparing mastectomy followed by prepectoral or subpectoral direct-to-implant breast reconstruction using the validated CSS. 19 It has previously been suggested that subpectoral reconstructed patients are more prone to experience impaired shoulder function compared to those reconstructed with the prepectoral technique. 6 7 9 This could not be confirmed in our study, which revealed no significant differences in shoulder function between the two reconstructed groups at baseline, nor at 3- and 12-month follow-up ( Table 2 ). Furthermore, no differences in CSS were found when comparing baseline to 12-month follow-up within each group, although a brief difference was found in the subpectoral group as well as in the modified CSS for the prepectoral group at 3-month follow-up ( Table 3 ). Our analysis showed a significant difference in pain in the subpectoral group at 12-month follow-up and in the PMM strength in both groups at 3-month follow-up, indicating the manipulation of the pectoralis muscle might be a contributing factor ( Table 5 ). Two prospective studies 7 9 and a retrospective study 6 focused on early functional outcomes. A study by Lee et al. aimed to investigate early functional recovery, including ROM, maximal muscle power, shoulder disability, and shoulder pain in women reconstructed by either of the two reconstructive techniques. They found that the prepectoral group experienced significantly less shoulder pain and shoulder disability at 2-week follow-up, but this difference was not seen at later follow-ups. Thus, both groups had recovered to the preoperative state at 6-month follow-up. They found no difference in muscle power or ROM at any given time during their trial. 7 A study by Cattelani et al. focused on the shoulder and arm impairment in women following reconstruction by prepectoral technique and the subpectoral technique without AMM support. This study found that the prepectoral group had faster functional recovery appearing which became evident from day 7 and persisted through day 30. The patients also reported less pain in the prepectoral group, but it is unclear whether this pain was shoulder-related. 9 The study by Caputo et al. assessed the shoulder function, including ROM and strength in women following pre- or subpectoral implant placement. They found statistically significant differences in active shoulder joint ROM in favor of the prepectoral group at 1-month follow-up; however, the difference was not present in all ROM planes and the subpectoral group was advised to rest their shoulder more in the first 2 weeks after surgery, which may have affected their ROM. 6 Contrary to these findings, the study by Lee et al. 7 found no evidence of affected muscle power or ROM at any given time during the follow-up period. All three of the mentioned studies focused on investigating the early phase of recovery. Our study population was not examined in the early phase of recovery, and therefore our results do not provide information about the patients' shoulder function earlier than 3 months after surgery. If we had assessed the shoulder function in our patients before 3 months, we might have found similar results to those presented in the three abovementioned studies. 6 7 9 Some studies have stated that lifting the PMM from its insertion affects the shoulder function. 10 12 This, combined with the findings of Lee et al., Cattelani et al., and Caputo et al., as well as our study, suggests that the dissection may impact the shoulder function primarily during the early phase of recovery when the muscle trauma is still recent. However, over time, it seems that the PMM regains its presurgery function, possibly combined with compensation by agonistic muscle. While Cattelani et al. and Caputo et al. had no functional follow-ups later than 1 month following surgery, 6 9 our results suggest that there is no difference in shoulder function from 3 months onward, which is in accordance with the results presented by Lee et al. 7 A study by Leonardis et al. assessed the long-term effects on shoulder function based on shoulder strength and stiffness. Patients were, on average, tested 20 months postsurgery. The study found that the strength and stiffness of the shoulder were significantly reduced in the plane of adduction in the subpectoral group ( *n* = 14) compared to the healthy age-matched control group ( *n* = 14); however, the difference was not present in other planes of motion. It is important to note that the study population was relatively small, the patients were compared to healthy controls rather than women reconstructed with prepectoral implant placement, and shoulder strength was not measured prior to surgery to provide a baseline for comparison. 10 A previous systematic review and meta-analysis analyzing postoperative outcomes, including shoulder function in women reconstructed by pre- and subpectoral implant placement, expressed the need for an RCT with a stronger baseline comparison examining postoperative complications, including pain and shoulder function. 12 Our study and Lee et al. 7 ensured baseline comparability regarding shoulder function; however, Caputo et al., 6 Cattelani et al., 9 and Leonardis et al. 10 did not. In our study, all patients had the shoulder function examined before surgery, as well as at 3 and 12 months after surgery. Furthermore, patient-related factors, surgical factors, and adjuvant treatments, including postoperative complications, showed homogeneity between our two groups, ensuring strong comparable baseline data, limiting the risk of possible patient-related confounders, several of which have been found to influence the surgical course. 2 This RCT is to the extent of our knowledge, the first to examine shoulder function by either of the two reconstructive techniques using the CSS. Additionally, patients enrolled in this study had a longer follow-up period compared to previous studies. 6 7 9 Our study was conducted as an RCT, and patients' allocation was concealed until surgery. However, blinding of patients or surgeons was not feasible after surgery. This may have introduced unconscious bias due to the awareness of the intervention received, potentially influencing the outcome. A limitation of our study is our small study population of 42 patients, thus restricting the generalizability of our findings. A larger sample size could allow for more detailed analysis; however, we do not believe this would affect the results. The sample size and power calculation of this study were based on the primary outcome, breast animation deformity of the original trial, 13 thus making it less accurate for this study and outcome. Another limitation of this RCT is the limited follow-up of 12 months, as we do not know whether shoulder function will change over time. Associations between impaired muscle function and radiotherapy have been described previously. 22 However, no patients included in this study received radiotherapy. Future studies should address the possible impact of radiotherapy on the PMM and the long-term shoulder function, with respect to the choice of reconstruction plane, as this could provide valuable insight.

In conclusion, we found no difference in shoulder function or PMM strength when comparing women reconstructed by prepectoral and subpectoral implant placement in direct-to-implant breast reconstruction, suggesting that concerns regarding impaired shoulder function should not influence the choice of pocket plane. However, in the subpectoral group, we did find a difference in pain when comparing baseline data to 12-month follow-up. Future studies should include a greater study population, with sample size calculations based on the shoulder function as the primary outcome and a follow-up period expanding beyond 12 months, as this will give a more comprehensive understanding of the shoulder function.
